# Supplementary material for: Prevalence and correlates of metabolic syndrome and its components in adults with psychotic disorders in Eldoret, Kenya
Source: PLoS One. 2021 Jan 11;16(1):e0245086. doi: 10.1371/journal.pone.0245086 (PMC7799838; doi:10.1371/journal.pone.0245086)
Supplement: Attachment — Submitted filename: Response to Reviewers.pdf [file pone.0245086.s002.pdf]

6TH OCTOBER 2020

THE EDITOR,

PLOS ONE JOURNAL

DR EDITH KWOBAH,

P.0 BOX ,30100,

ELDORET KENYA

eckamaru@gmail.com

TEL +254721763412

RE: MANUSCRIPT RESUBMISSION

We thank you for the review of a manuscript titled “Prevalence and Correlates of Metabolic Syndrome and its Components Among Adults with Psychotic Disorders in Eldoret, Kenya”.

We have addressed all the concerns raised as follows:

1. **Methods (Page 5)– please clarify whether the inclusion criteria for the study required psychotic symptoms (for example, bipolar disorder with psychotic features)**
   The inclusion criteria required that the participant fulfill the criteria for a psychotic disorder (i.e., bipolar mood disorder with psychotic features, schizophrenia or schizoaffective disorder according to DSM-5 criteria). This is now clarified.

2. **Methods (Page 6) – please add details on whether the blood glucose levels were fasting”**
   We have clarified that we used non fasting glucose and lipids. This is listed as a limitation.
3. **Methods (Page 6) – Please add details on which definition/criteria for Metabolic Syndrome was used for this study.**
   We have clarified that we used the Expert Panel on Detection, Evaluation, and Treatment of High Blood Cholesterol in Adults (Adult Treatment Panel III) to define Metabolic Syndrome, but with a random blood glucose equivalent for the fasting glucose component.

4. **Methods (Page 6) – Please clarify how non-normally distributed variables were handled.**
   We reassessed our data and found that normality was not present, so we used the nonparametric Wilcoxon rank-sum test in place of the t-test (*Table 2).*
5. **Methods (Page 6) – Please clarify if there was adjustment for multiple comparisons.**
   We have clarified that to account for multiple testing, we did a Bonferronni correction adjusting the significance level to 0.05/9= 0.005 instead of 0.05 (*Table 2).*
6. **Discussion (Page 12) – While it may not be possible to fully explain why this study finding differed from other work in South Africa and Uganda, please expand upon whether the age, sex, antipsychotic treatments were similar to this study sample. A deeper examination of the differences in this population from South Africa (the source of most African studies on this subject) would establish why these studies should not be lumped together.**
   We have clarified that these studies are similar in having a young aged population, but differed in that majority of the participants in previous work in Uganda and South Africa were on first generation antipsychotics.
7. **Discussion (Page 12) – Please clarify in the third paragraph discussing the obesity rates whether the comparison is with general population samples or with participants with psychotic disorders. Also, the varying rates across country are not fully explored – please add further discussion**
   This section has been modified to focus on comparisons with studies of patients with mental illness.
8. **Discussion (Page 12-13) – given the higher rates of obesity among women, it would be helpful to clarify if women and men had differences in demographic, medication, or other clinical factors that might be related.**
   There were no gender differences between men and women in terms of their demographic, medical or clinical factors that could explain the excess obesity among women. This point is now added.
9. **Discussion (Page 14, 2nd paragraph) – please correct the typos in the second sentence "a 2.4% diabetes.")**

   This has been corrected to read "reported a 2.4% prevalence of diabetes".

10. **Discussion (Page 15) – the high rates of olanzapine usage are an important contributor to the metabolic health of these patients. Please clarify if the other African studies of metabolic health had similarly high rates of olanzapine or other SGAs with high metabolic risk.**

    We have expanded this to indicate that in the 3 prior African studies most patients were on first generation antipsychotic, where as in our study the majority were on olanzapine. We have also added a brief discussion of this point, noting the advantages but potential disadvantages of donated medication.

11. **Discussion (Page 15) – another key point for clarification in whether olanzapine does/does not contribute to metabolic health is whether there were demographic differences in those who were on olanzapine vs. another antipsychotic (age, sex, education level, age of onset) as these factors may confound this relationship.**

    We have clarified that there was no sex or education differences between clients on olanzapine and those who were not on olanzapine. Even after adjusting for age, sex and education level the relationship between olanzapine and various components of metabolic syndrome were still not significant. However, the low numbers of participants not on olanzapine makes it difficult to draw definitive conclusions, a point also noted in the discussion.

12. **Discussion – Please expand on the screening practices and treatment gaps within this particular treatment setting. Rates of monitoring of metabolic health for patients on olanzapine vary by treatment setting/provider – it would be helpful to not whether this is a common practice in this setting.**

    This has been expanded to indicate that currently only body weight and blood pressure are taken and that there is no clear protocol for systematic measuring of blood glucose or lipid profile. We discuss the need for this to be put in place.

13. **Discussion – Deinstitutionalization in Western countries impacted mental and physical health outcomes for patients with psychotic disorders. It would be interesting to discuss whether such practices have occurred in Kenya and whether that may influence the health outcomes in this population.**

    In this part of Kenya, there is not a history of institutionalizing large numbers of psychotic patients for long periods of time. The inpatient facility studied provides short-term care for patients from a large geographic region, who are then discharged to their communities. Our discussion now indicates that in these settings, better monitoring of CVD risk factors is needed.

14. We have clarified that Written Consent was obtained from all participants.

We look forward to a positive response.

Sincerely,

EWK

Edith Kwobah.
