# Supplementary material for: Prevalence and correlates of metabolic syndrome and its components in adults with psychotic disorders in Eldoret, Kenya
Source: PLoS One. 2021 Jan 11;16(1):e0245086. doi: 10.1371/journal.pone.0245086 (PMC7799838; doi:10.1371/journal.pone.0245086)
Supplement: Attachment — Submitted filename: Responce to Reviewers_.docx [file pone.0245086.s003.docx]

25TH NOVEMBER 2020

THE EDITOR,

PLOS ONE JOURNAL

DR EDITH KWOBAH,

P.0 BOX 3, 30100,

ELDORET KENYA

TEL +254721763412

**RE: RESPONSE TO REVIEWERS**

We thank you for the review of our manuscript titled “Prevalence and Correlates of Metabolic Syndrome and its Components Among Adults with Psychotic Disorders in Eldoret, Kenya”.

We have responded to each of the reviewers comments comments as follows:

1. **Abstract – Results section: Please clarify that the marital status variable was widowed/separated/divorced (compared to never married and married groups). These were groupings show in Table 1.**

We would like to clarify that there were 3 categories of marital status, married, never married, and Widow/Separated / divorced as shown on tables 1 and 3.

2. **Results – please note the age range of the participants in this study, to help clarify the relationship between older age and metabolic syndrome**.

We have clarified in the results that the age range was 18-67 for patients and 18-69 for controls

3. **Results – please clarify whether the obesity findings in Table 4 were based on BMI or abdominal obesity. The BMI/abdominal obesity findings in the discussion should be mentioned in the results section as well.**

We have clarified that elevated BMI was used to define obesity in Table 4.We would also like to clarify that the prevalence of BMI and abdominal obesity as described in the discussions are captured in Figure 1.

4. **Discussion – the finding that female gender was associated with decreased odds of metabolic syndrome but increased odds of obesity are puzzling and warrant further discussion.**

We agree that indeed this is quite unexpected. The difference may lie in other risk factors of metabolic syndrome such as genetics, and environmental factors. We have expanded this discussion to indicate that this would warrant further investigation.

5. **Discussion- the discrepancy in gender findings on metabolic syndrome from the current study and South African study are also puzzling. Were there age/other demographic factor differences between the two cohorts? What were the findings regarding obesity in the two groups?**

We agree that the differences Kenyan and South Africa studies are quite unexpected given that the two studies were quite similar in terms of age (mean age of 33 in Kenya and 37 in South Africa) and also in prevalence of obesity (45% in Kenya and 46 % In South Africa.

6. **Discussion – additional interpretation/synthesis would be helpful to understand why the present study would differ from the Western Kenya study. Are the two groups of participants comparable in terms of demographic features or treatments?**

We have adjusted the statement to clarify that the findings of the Health and Demographic Surveillance System (HDSS) study in Western Kenya region demonstrates the excess rates of dyslipidaemia among patients compared to the general population.

7. **Discussion – the low numbers of participants on agents other than olanzapine make it difficult to truly link olanzapine use to metabolic problems. It may be helpful to emphasize the finding that dosage of olanzapine was not linked to metabolic syndrome or other cardio metabolic abnormalities**.

We have emphasised that the lack of association between olanzapine and metabolic syndrome and its components is quite unexpected. We have also reconsidered and removed the statement about the low number of patients on olanzapine making it difficult to truly link it to metabolic syndrome - under limitations- this is because in the first place olanzapine was not associated with either the metabolic syndrome or any of its components.

8. **Discussion – please clarify what is indicated by “human resources for mental healthcare” – would those be increasing the staffing of mental health clinics or other types of support?**

We have clarified that human resources for mental health care refers to increasing the number of staff whose mandate is to provide mental health services- this includes nurse, psychiatrists, social works, psychologists and occupational therapists.

We look forward to a favourable response and are happy to respond to any other clarifications.

Sincerely,

Edith Kwobah,

Corresponding Author.
